# Solamargine Inhibits Prostate Cancer Cell Growth and Enhances the Therapeutic Efficacy of Docetaxel via Akt Signaling

**DOI:** 10.1155/2022/9055954

**Published:** 2022-03-10

**Authors:** Jianchao Ge, Pengyu Wang, Hangbin Ma, Jun Zhang

**Affiliations:** Department of Urology, Shanghai Fifth People's Hospital, Fudan University, Shanghai, China

## Abstract

Prostate cancer (PCa) has become a leading cause of cancer-associated incidence and mortality in men worldwide. However, most primary PCas relapse to castration-resistant PCa (CRPC) after androgen deprivation treatment. The current treatment for CRPC is based on chemotherapeutic drugs such as docetaxel, while the development of chemoresistance and severe side effects limit the therapeutic benefit. Solamargine, a natural alkaloid isolated from a traditional Chinese herbal medicine known as *Solanum nigrum*, exhibits antitumor activity in various human cancers. In this study, we demonstrated that solamargine substantially inhibited CRPC cell growth in a dose-dependent manner through the suppression of phosphoinositide 3-kinase (PI3K)/Akt signaling. Moreover, solamargine exhibited significant antitumor effects in mouse xenograft models. Bioinformatics analysis of docetaxel-resistant PCa cells indicated that the PI3K/Akt pathway mediated the chemoresistance of CRPC. Furthermore, solamargine significantly enhanced the efficacy of docetaxel in PCa cells. These results reveal the therapeutic potential of solamargine against human PCa.

## 1. Introduction

Prostate cancer (PCa) is the second leading cause of cancer-related death in men worldwide [1]. Because of the essential role played by androgen receptor (AR) signaling in the tumorigenesis of PCa [2], most patients with primary PCa receive androgen deprivation treatment (ADT) as initial therapy [3]. Although ADT achieves a desired response in the early stage of PCa [4], almost all patients relapse to castration-resistant PCa (CRPC) within 18–24 months, which is the major concern in PCa treatment [5]. Thus, patients are treated with chemotherapy such as docetaxel, which may cause severe adverse effects and impair the quality of life [6]. Novel therapeutic strategies are urgently needed for PCa treatment, particularly CRPC.

In this study, we demonstrated the antiproliferative effects of solamargine on CRPC cells in vitro and in vivo. Furthermore, solamargine substantially inhibited the protein level of phosphorylated (p-Akt). The phosphoinositide 3-kinase (PI3K)/AKT signaling pathway, which is aberrantly activated in approximately 60% of PCa patients [7], is associated with adverse clinicopathological variables and decreased disease-specific survival [8]. Akt phosphorylation, which activates downstream transcription factors and target genes, is commonly recognized as a key driver of prostate tumorigenesis [9]. Aberrantly activated Akt has long been identified as an attractive therapeutic target, and several Akt inhibitors are currently under investigation in clinical trials [10–12].

Notably, Akt phosphorylation has a causal role in regulating cell viability and mediating chemoresistance in breast cancer [13]. In addition, targeting the PI3K/Akt signaling pathway alleviates ovarian cancer chemoresistance and reverses the epithelial-mesenchymal transition [14]. Moreover, the PI3K/AKT pathway has been linked to both tumorigenesis and resistance to ADT in PCa [15]. We demonstrated the crosstalk between PI3K/Akt and chemoresistance by performing enrichment analysis of docetaxel-resistant CRPC cells, which indicated the PI3K axis as a rational co-target for combination therapy in CRPC. Solamargine in combination with docetaxel led to a significant decrease in cell viability, compared with solamargine or docetaxel alone.

In many cases, combination therapies enhance treatment efficacy and delay the onset of side effects, serving as a significant option for the treatment of multiple cancers such as PCa. Clinical trials have demonstrated limited single-agent efficacy in CRPC [16, 17]. These results reveal a common limitation of targeted treatments: specific inhibition of a single therapeutic target may trigger compensatory mechanisms and activation of other signaling or parallel growth pathways [18, 19]. The current hypothesis indicates that co-targeting a compensatory bypass is required for the treatment of PCa cancer.

In conclusion, our results demonstrate the antitumor activity of solamargine as a single agent and in combination with docetaxel. In addition, expression analysis revealed the upregulation of PI3K/AKT target genes upon docetaxel resistance, suggesting a compensatory survival and growth mechanism that requires the targeting of both pathways for optimal therapeutic efficacy. Taken together, the findings in this study provide a potential therapeutic strategy to target CRPC and chemoresistant PCa.

## 2. Materials and Methods

### 2.1. Chemicals and Reagents

Solamargine (HY-N0069, purity ≥ 98%) and docetaxel (RP-56976, purity ≥ 99%) were purchased from MedChemExpress (Monmouth Junction, NJ, USA). Drugs were dissolved in dimethyl sulfoxide (DMSO; Sigma-Aldrich, St. Louis, MO, USA).

### 2.2. Cell Culture

The human CRPC cell lines PC3 and DU145 were purchased from the American Type Culture Collection (Manassas, VA, USA). The 293T cell line was kindly provided by the Stem Cell Bank, Chinese Academy of Sciences (Shanghai, China). DU145 and 293T cells were cultured in a Dulbecco's modified Eagle medium. PC3 cells were cultured in a RPMI-1640 medium supplemented with 10% fetal bovine serum (Gemini, Woodland Hills, CA, USA), 1% HEPES (Corning Inc., NY, New York, USA), and 1% penicillin/streptomycin (Gibco, Grand Island, NY, USA). All cells were maintained in a 5% CO_2_ humidified incubator at 37°C.

### 2.3. Plasmids and Transfection

Constitutively active Akt (i.e., myristoylated Akt) was cloned into the pLVX-IRES-Puro vector (632183; Clontech Laboratories, San Jose, CA, USA). Plasmids were transduced into HEK293T cells using PEI 25K (23966-1; Polysciences, Warrington, PA, USA), in accordance with the manufacturer's instructions. Stable transformants of DU145 and PC3 cells were isolated in complete medium supplemented with puromycin (5 *μ*g/mL; Sigma) for 3 days.

### 2.4. Cell Viability and Proliferation Assay

Cells were digested and seeded in 96-well plates (1000 cells per well). Cell growth was detected by the Cell Counting Kit-8 (CCK-8) assay (CK04; Dojindo, Kumamoto, Japan) at the indicated time points, in accordance with the manufacturer's instructions. Then, 100 *μ*L of complete medium supplemented with the10% CCK-8 reagent were added to each well and incubated for approximately 3 h at 37°C. The absorbance values at 450 nm were detected using a microplate reader (Tecan, Mechelen, Belgium). Cell viability (%) of the experimental group was calculated as the percent of the control group. The colony formation assay was performed in accordance with the method established by Ge et al. [20].

### 2.5. Wound Healing Assay

Approximately, 1 × 10^5^ PC3 and DU145 cells were seeded in a 12-well plate and incubated until confluence was reached; a linear scratch was then made using a sterile P200 pipette tip. The cells were washed three times with phosphate-buffered saline, and then a fresh culture medium containing solamargine (DMSO as the control) was added. Images were acquired from 0 to 24 h using a phase-contrast microscope. The wound closure gap was determined by dividing the area by the length of the scratch, then comparing with the value in DMSO-treated group.

### 2.6. Flow Cytometry

Approximately, 5 × 10^5^ PC3 or LNCaP cells were seeded in a 6-well plate and cultured overnight. Cells were treated with solamargine at the indicated concentration for 24 h. Cells were harvested and resuspended in 100 *μ*L of 1 × binding buffer (included in the Annexin V-FITC/PI Apoptosis Detection Kit; Vazyme Biotech, Nanjing, China), then stained with 5 *μ*L of Annexin V-FITC and propidium iodide (PI) in accordance with the manufacturer's instructions. After 10 min incubation at room temperature in the dark, 400 *μ*L of 1 × binding buffer was added, and the apoptosis rates of PC3 and LNCaP cells were detected by flow cytometry.

### 2.7. Western Blot Analysis

Cells were harvested and solubilized in a RIPA lysis buffer supplemented with phenylmethylsulphonyl fluoride and PhosSTOP Phosphatase Inhibitor Cocktail (Roche, Monza, Italy). Aliquots were loaded and separated by sodium dodecyl sulfate-polyacrylamide gel electrophoresis, then electrotransferred to polyvinylidene fluoride membranes. The membranes were blocked in 5% bovine serum albumin for 1 h, then incubated with primary antibodies at 4°C overnight. Subsequently, the membranes were incubated with secondary antibodies for 1 h, after three washes with 1 × Tris-buffered saline with 0.1% Tween 20. The protein signal density was detected using the FluorChemE imager (ProteinSimple, San Jose, CA, USA). The primary antibodies used in the study were as follows: phospho-Akt (Ser473) (#4060S; Cell Signaling Technology, Danvers, MA, USA), Akt (# 4691S; Cell Signaling Technology), and *β*-actin (sc-47778; Santa Cruz Biotechnology).

### 2.8. Animal Experiments

Approximately, 1 × 10^6^ PC3 cells resuspended in phosphate-buffered saline were subcutaneously injected into 6-week-old male nude mice. Mice were treated with DMSO or solamargine when the implanted tumor size reached approximately 50 mm^3^. After approximately 8 weeks, all mice were sacrificed; the tumors were dissected and weighed. The xenografts were fixed in formalin, then paraffin-embedded for immunohistochemistry (IHC) and hematoxylin-eosin staining. All procedures were approved by the Institutional Animal Care and Use Committee of Shanghai Veterinary Research Institute (Shanghai, China).

### 2.9. IHC Staining

The xenograft slides were deparaffinized in xylene solution and rehydrated in graded ethanol. Then, tissue sections were incubated in 3% hydrogen peroxide for 10 min and immersed in a citrate buffer (pH 6.0) at 95°C for 20 min. The sections were cooled, then blocked in the preferred blocking solution for 1 h at room temperature, washed, and subsequently incubated with primary antibodies at 4°C overnight. The following antibodies were used for IHC: phospho-Akt (Ser473) (#4060S; Cell Signaling Technology) and Ki67 (A2094; ABclonal Technology, Woburn, MA, USA).

### 2.10. Gene Set Enrichment Analysis

Gene expression datasets of docetaxel-resistant PC3 and DU145 cells were downloaded from National Cancer for Biotechnology Information-Gene Expression Omnibus (https://www.ncbi.nlm.nih.gov/geo/). Gene Set Enrichment Analysis was conducted using software provided by the Broad Institute (https://www.broadinstitute.org/gsea/index.jsp). The permutation type was “gene set,” and the genes were ranked by Pearson's correlation.

### 2.11. Statistical Analyses

Statistical analyses in this study were performed using GraphPad Prism software (version 7; GraphPad Software, La Jolla, CA, USA). Quantitative data obtained from experiments were analyzed by Student's *t-*test and presented as means ± standard deviations. *P* < 0.05 was considered statistically significant. ^*∗*^*P* < 0.05,  ^*∗∗*^*P* < 0.01, and  ^*∗∗∗*^*P* < 0.001.

## 3. Results

### 3.1. Growth Inhibitory Activity of Solamargine in CRPC Cells

**Figure 1:**
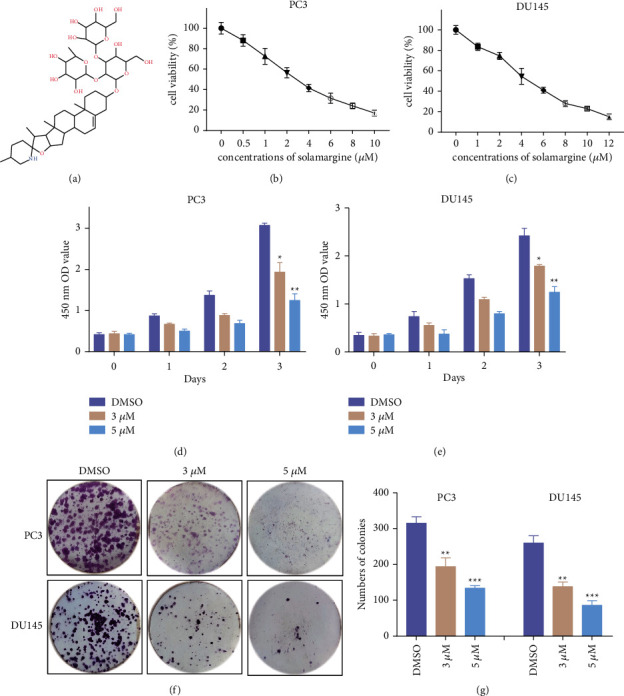
Effect of solamargine on cell viability of CRPC cells. (a) Chemical structure of solamargine. (b) and (c) PC3 and DU145 cells were treated with the indicated concentrations of solamargine (SM) for 48 h cell viability was determined by CCK8 assay. (d) and (e) PC3 and DU145 cells were treated with 3 *μ*M and 5 *μ*M solamargine, and then the cell viability was evaluated at the indicated time points. (f) Colony formation assay was employed to test the long-term cell proliferation of PC3 and DU145 cells after solamargine treatment for about two weeks. (g) Quantitative histograms of colony formation assay are shown. (Values represent mean ± SD.  ^*∗*^*P* < 0.05,  ^*∗∗*^*P* < 0.01, and  ^*∗∗∗*^*P* < 0.001 versus control).

CRPC cells were treated with DMSO (control) or solamargine (0.5–10 *μ*M for PC3 and 1–12 *μ*M for DU145), and the cell viability was detected at 48 h post-treatment. For both CRPC cell lines, solamargine demonstrated a dose-dependent inhibition of cell proliferation (Figures 1(b) and 1(c)). The IC_50_ values calculated from the given dose curves were 3.25 *μ*M in PC3 cells and 4.52 *μ*M in DU145 cells. Then, we treated the CRPC cells with 3 and 5 *μ*M to determine whether solamargine suppresses cell growth in a time-dependent manner. Cell proliferation was analyzed using the CCK-8 assay; the results revealed that solamargine significantly reduced the proliferation rate of PCa cells (Figures 1(d) and 1(e)). Colony formation assays yielded similar results, in which solamargine led to significant reductions in cell colony numbers relative to controls (Figures 1(f) and 1(g)). The results of the CCK-8 and colony formation assays in LNCaP cells are presented in Figures S1A and S1B. Collectively, these results indicate that solamargine potently inhibits the growth of CRPC cells.

### 3.2. Solamargine Suppresses CRPC Cell Migration and Induces Apoptosis

**Figure 2:**
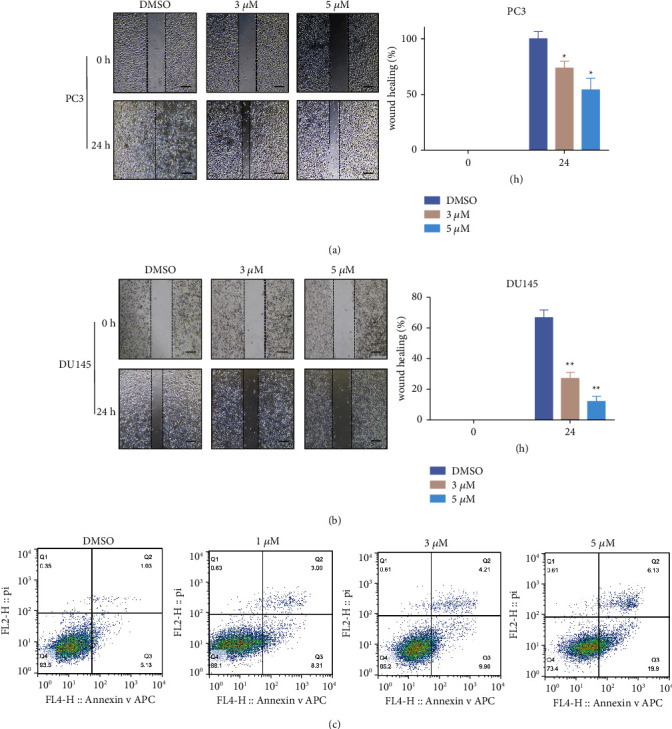
Solamargine induced apoptosis and inhibited migration ability of CRPC cell lines. (a) and (b) Effect of solamargine on the migration ability of CRPC cells. PC3 and DU145 cells were wounded using a 200 *μ*L micropipette tip and then incubated with or without solamargine. Cell images were taken at 0 and 24 h. The dotted lines show the area where the scratch wound was made. Scale bar: 100 *μ*m. Quantitative data are presented as means ± SD of three independent experiments. (Values represent mean ± SD. ^*∗*^*P* < 0.05 and ^*∗∗*^*P* < 0.01 versus control). (c) PC3 cells were treated with different concentrations of solamargine for 24 h and then stained using Annexin V-FITC/PI. Cell apoptosis rates were determined using flow cytometry.

To evaluate the effects of solamargine on the migration capacity of CRPC cells, PC3 and DU145 cells were subjected to different concentrations of solamargine for 24 h; their abilities to migrate were measured using the wound healing assay. As shown in Figures 2(a) and 2(b), solamargine inhibited the migration of PC3 and DU145 cells compared to the control. The antimigratory activity of solamargine was also observed in LNCaP cells (Figures S1C and S1D). Then, we investigated the apoptosis phenotype induced by solamargine in PC3 and LNCaP cells. Flow cytometry analysis showed that solamargine induced PCa cell apoptosis in a dose-dependent manner (Figures 2(c) and S1E).

### 3.3. Solamargine Inhibits CRPC Cell Proliferation through Akt Signaling

**Figure 3:**
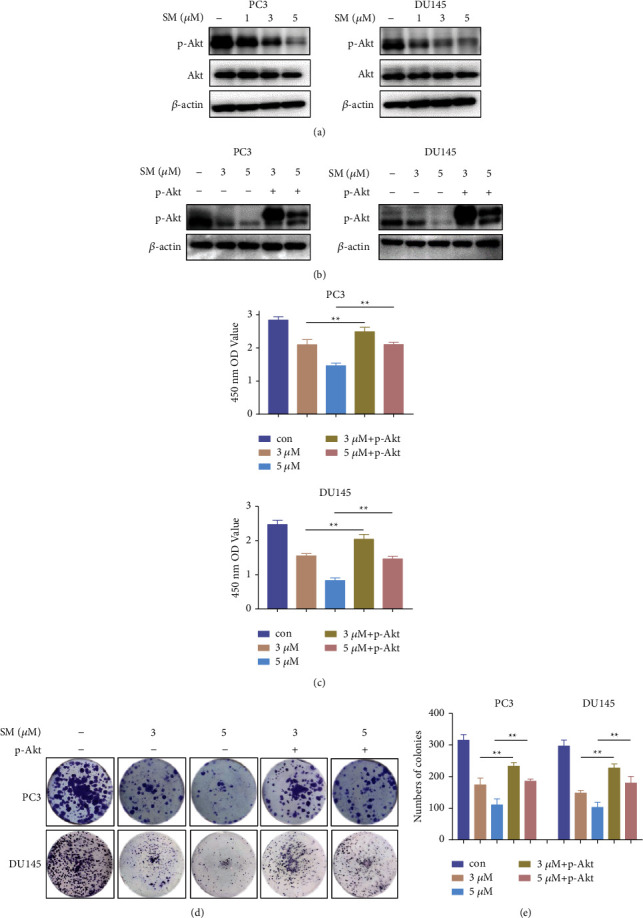
Solamargine impaired CRPC cell growth through Akt signaling. (a) PC3 and DU145 cells were treated with the indicated concentrations of dimethyl sulfoxide (DMSO) or solamargine (SM), and the protein levels of phosphorylated Akt (p-Akt) and total Akt were measured using Western blotting. (b) Constitutively active Akt plasmid was transfected into PC3 cells, and the expression level of p-Akt was detected using Western blotting. (c) Cell viability was evaluated by CCK-8 assay. (d) Solamargine-treated cells with or without Akt overexpression were examined by colony formation assay. (e) The numbers of colonies for each group were counted. (Values represented mean ± SD. ^*∗*^*P* < 0.05 and ^*∗∗*^*P* < 0.01 versus control.).

AR and PI3K/AKT pathways are considered the two most crucial growth pathways in PCa tumorigenesis and progression. Because solamargine significantly inhibited the proliferation of CRPC cells, we examined whether the inhibition of CRPC cell proliferation by solamargine was derived from Akt suppression. Solamargine treatment (24 h) caused a dose-dependent decrease in the p-Akt protein level in CRPC cells compared to the control group (Figure 3(a)). In addition, solamargine reduced the abundance of p-Akt in androgen-dependent LNCaP cells (Figure S1F).

To confirm that solamargine suppresses CRPC cell growth through Akt signaling, we transfected myristoylated Akt (Myr-Akt) plasmids into PC3 cells to explore whether Akt overexpression could counteract the antiproliferation effects of solamargine. The expression levels of p-Akt were evaluated by Western blotting (Figure 3(b)); the cell growth was measured using CCK-8 and colony formation assays. Ectopic expression of Myr-Akt substantially alleviated the inhibition of solamargine-treated CRPC cells (Figure 3(c)). Moreover, the colony formation abilities of solamargine-treated cells were restored by Akt overexpression (Figures 3(d) and 3(e)). These results indicate that solamargine causes inhibition of PCa cell proliferation through the suppression of Akt signaling.

### 3.4. Solamargine Suppresses the Growth of CRPC Tumor Xenografts

**Figure 4:**
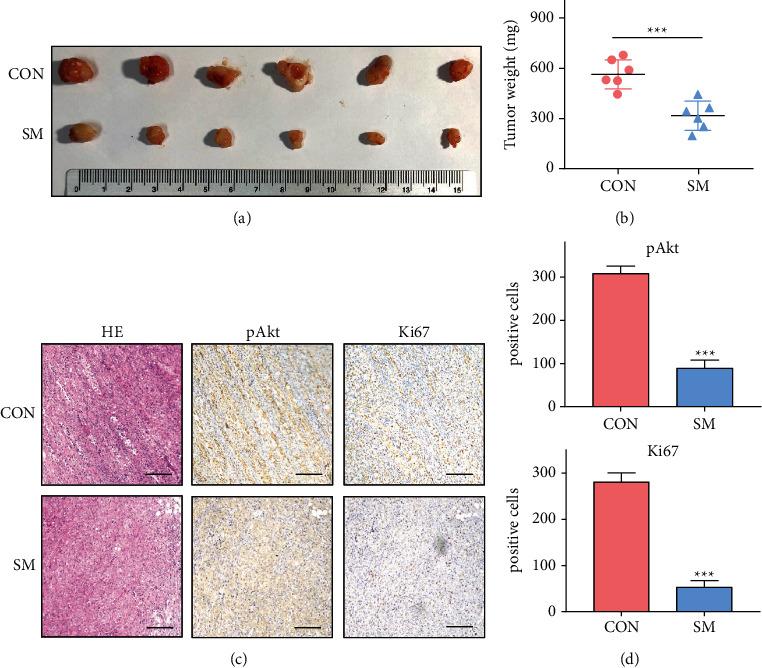
Solamargine impeded PCa tumor growth in vivo. (a) PC3 cells (1 × 106) were subcutaneously injected into nude mice (*n* = 6). Mice were treated with dimethyl sulfoxide (DMSO) or solamargine (SM) when the xenograft size reached 50 mm^3^. Dose schedules were DMSO or solamargine (5 mg/kg, intraperitoneal, once per 2 days for 4 weeks). Mice were sacrificed and tumor volume is shown. (b) The weights of the xenografts are shown. Error bars represent mean ± standard deviation (Mann-Whitney test; *n* = 6; ^*∗∗∗*^*P* < 0.001). (c) Representative images of hematoxylin-eosin (HE) staining and IHC staining in xenografts (scale bar: 100 *μ*m). (d) p-Akt and Ki67 expression is presented as the number of positive cells (Mann–Whitney test).

To further confirm the antiproliferative effects of solamargine on CRPC cells *in vivo*, PC3 cells were subcutaneously implanted into 6-week-old male nude mice to establish mouse xenograft models. After 8 weeks, the mice were sacrificed and the xenografts were extracted for further investigation. Neither treatment induced any obvious side effects, such as diarrhea or weight loss. Tumors of the solamargine-treated group grew more slowly (Figure S1G); their final weight and volume were lower than the weight and volume in the control group. In addition, IHC staining analysis of the xenograft tissues revealed that solamargine significantly reduced p-Akt and Ki67 expression levels, indicating impaired tumor cell viability (Figures 4(c) and 4(d)). These results demonstrate that solamargine significantly inhibited CRPC cell growth *in vivo*.

### 3.5. Synergistic Antitumor Effect of Solamargine in Combination with Docetaxel on CRPC Cells

**Figure 5:**
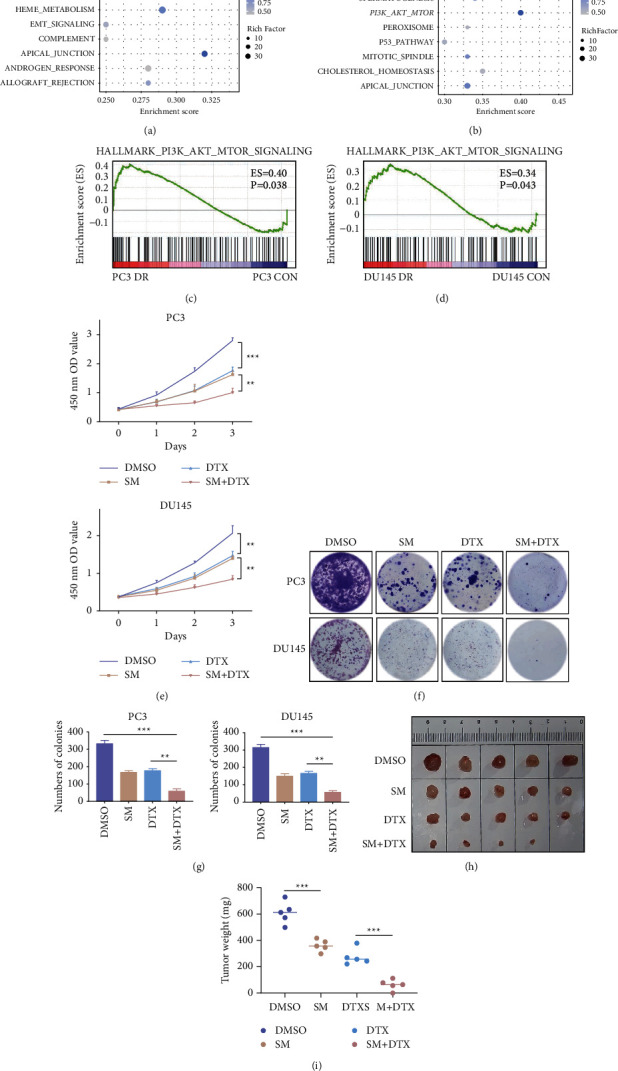
Combination of solamargine and docetaxel results in increased inhibition of CRPC cells. (a) and (b) Bubble plots of GSEA results of PC3 and DU145 cells. (c) and (d) Gene expression profiles of CRPC docetaxel-resistant cells based on the PI3K/Akt/mTOR signaling gene set versus control. (e) PC3 and DU145 cells were treated with or without solamargine (3 *μ*M) and docetaxel (1 nM). Cell viability was detected using CCK8 assay at the indicated time points. (f) Effects on colony-forming abilities of solamargine and docetaxel. (g) Histograms show the number of colonies. (Values represented mean ± SD. ^*∗∗*^*P* < 0.01, and ^*∗∗∗*^*P* < 0.001). (h) The PC3 xenografts were established in nude mice. Mice treated with dimethyl sulfoxide (DMSO), solamargine alone, docetaxel alone, double combinations when the size of xenograft reached 50 mm^3^. Dose schedules were solamargine (5 mg/kg, intraperitoneal, once per 2 days for 4 weeks), and docetaxel (5 mg/kg, intraperitoneal, once a week for 4 weeks). (i) The weight of the xenograft is shown. Error bars represent mean ± standard deviation (Mann-Whitney test; *n* = 5; ^*∗∗∗*^*P* < 0.001).

Docetaxel is the current first-line chemotherapy for CRPC, while chemoresistance and adverse reactions are unavoidable. To explore new methods to achieve optimal efficacy and minimize side effects, we downloaded the gene expression profiles of docetaxel-resistant PCa cells from Gene Expression Omnibus (GSE158494). Expression analysis was performed to investigate possible signaling pathways involved in chemoresistance (Figures 5(a) and 5(b)). The PI3K/Akt pathway was significantly enriched in both PC3 and DU145 docetaxel-resistant cells compared to the control group (Figures 5(c) and 5(d)). These data indicate that the PI3K/Akt pathway represents a key target for addressing rapid chemoresistance in CRPC.

Considering that solamargine significantly suppresses the expression of p-Akt, we examined whether the combination of solamargine and docetaxel could synergistically inhibit CRPC proliferation. The viabilities of PC3 and DU145 cells treated with solamargine and/or docetaxel were evaluated using the CCK-8 assay (Figure 5(e)). The results of the colony formation assay further confirmed the synergistic effects of solamargine and docetaxel (Figures 5(f) and 5(g)). Furthermore, the combination of docetaxel and solamargine resulted in a more profound inhibition of CRPC cell growth *in vivo* than did either drugs alone did. The control group xenografts (DMSO) were larger and heavier than xenografts in the solamargine-or docetaxel-treated groups. Taken together, these insights may help to develop new rational therapies for PCa.

## 4. Discussion

Docetaxel is the current first-line chemotherapy for CRPC [21]. Although docetaxel-based chemotherapy has significantly improved the overall survival of CRPC patients, durable responses are uncommon [22]. Furthermore, high doses of docetaxel induce significant toxicity and may cause adverse reactions such as neutropenia, alopecia, and nausea [23]. In addition, chemotherapy resistance has become a major cause of mortality in PCa patients and a major clinical challenge, highlighting the need to co-target compensatory pathways for treating PCa. A nontoxic agent that enhances the efficacy of docetaxel would reduce the dose of docetaxel and potentially improve prognosis.

Targeted cancer therapies provide the opportunity for personalized medicine tailored to the molecular characteristics of tumors. In PCa, the AR and PI3K/Akt pathways are considered the major drivers of tumor growth and progression. Several levels of crosstalk between the AR and PI3K/Akt pathways have been reported [24]. ADT targeting the AR axis is commonly used for primary PCa, while tumors eventually progress to CRPC [25]. Aberrant activation of the PI3K/Akt pathway, an essential regulator of cellular functions such as cell growth and proliferation, has been widely identified in many cancers including PCa [26]. In addition, Akt signaling participates in mediating chemoresistance in cancer cells [27].

In most instances, increased Akt signaling is correlated with reduced sensitivity to endocrine therapy or receptor tyrosine kinase inhibitors [28]. Therefore, an Akt inhibitor may be particularly useful for PCa with Akt activation. Currently, Akt inhibitors have shown significant efficacy in many preclinical models; they exhibit synergistic anticancer activity when combined with other therapeutic agents [29]. The combination of Akt inhibitors and docetaxel substantially prolonged the overall survival of CRPC patients in a phase II clinical trial [30].

Plant compounds are considered major sources of new drugs, and a large number of herbal products have been studied for antitumor activity [31]. *Solanum nigrum* is a widely used traditional Chinese medicine in clinical practice because of its anti-inflammatory and antitumor effects [32]. Solamargine, a steroidal alkaloid derived from *S. nigrum*, exhibits therapeutic activities in several cancers [33]. For example, solamargine inhibits gastric cancer progression by suppressing the mitogen-activated protein kinase pathway [34]. In addition, the combination of solamargine and metformin enhances the growth inhibition of PCa cells [35]. However, the detailed molecular mechanism underlying the inhibition of cancer cell proliferation by solamargine remains unknown.

In this study, we demonstrated the antiproliferative effect of solamargine on PCa both *in vitro* and *in vivo*. Furthermore, the suppression of solamargine is attributed to the inhibition of the PI3K/Akt signaling pathway. Considering that solamargine significantly reduces the p-Akt protein expression, we investigated whether the combination of solamargine with docetaxel has synergistic antitumor effects. The combination treatment exhibited a better antiproliferation effect on CRPC cells than solamargine or docetaxel alone did. Additional work is needed to investigate the specific downstream targets of PI3K/Akt suppressed by solamargine, thus providing a better understanding of the signaling pathways involved in the transformation of CRPC. In addition, it remains unknown whether solamargine can delay the development of chemoresistance in PCa cells or xenograft models.

## 5. Conclusions

In summary, this study demonstrated that solamargine suppresses CRPC cell proliferation both in vitro and in vivo. Furthermore, solamargine enhances the efficacy of docetaxel by inhibiting the PI3K/Akt pathway. These results provide new insights into the research and development of valid therapeutic applications for PCa.

## Data Availability

The datasets supporting the findings of this study are indicated in the article. Data will be made available on reasonable request.

## Abbreviations

AR: Androgen receptor
CCK8: Cell Counting Kit-8
CRPC: Castration-resistant prostate cancer
DMSO: Dimethyl sulfoxide
GSEA: Gene set enrichment analysis
IHC: Immunohistochemistry
p-Akt: Phosphorylated Akt
PCa: Prostate cancer
SM: Solamargine.

## Supplementary Materials

Supplementary Materials Figure S1: phenotype of AR-positive LNCaP cells induced by solamargine.
