# Supplementary figures and images for: Solamargine Inhibits Prostate Cancer Cell Growth and Enhances the Therapeutic Efficacy of Docetaxel via Akt Signaling
Source: J Oncol. 2022 Mar 10;2022:9055954. doi: 10.1155/2022/9055954 (PMC8930254; doi:10.1155/2022/9055954)

Figure S1

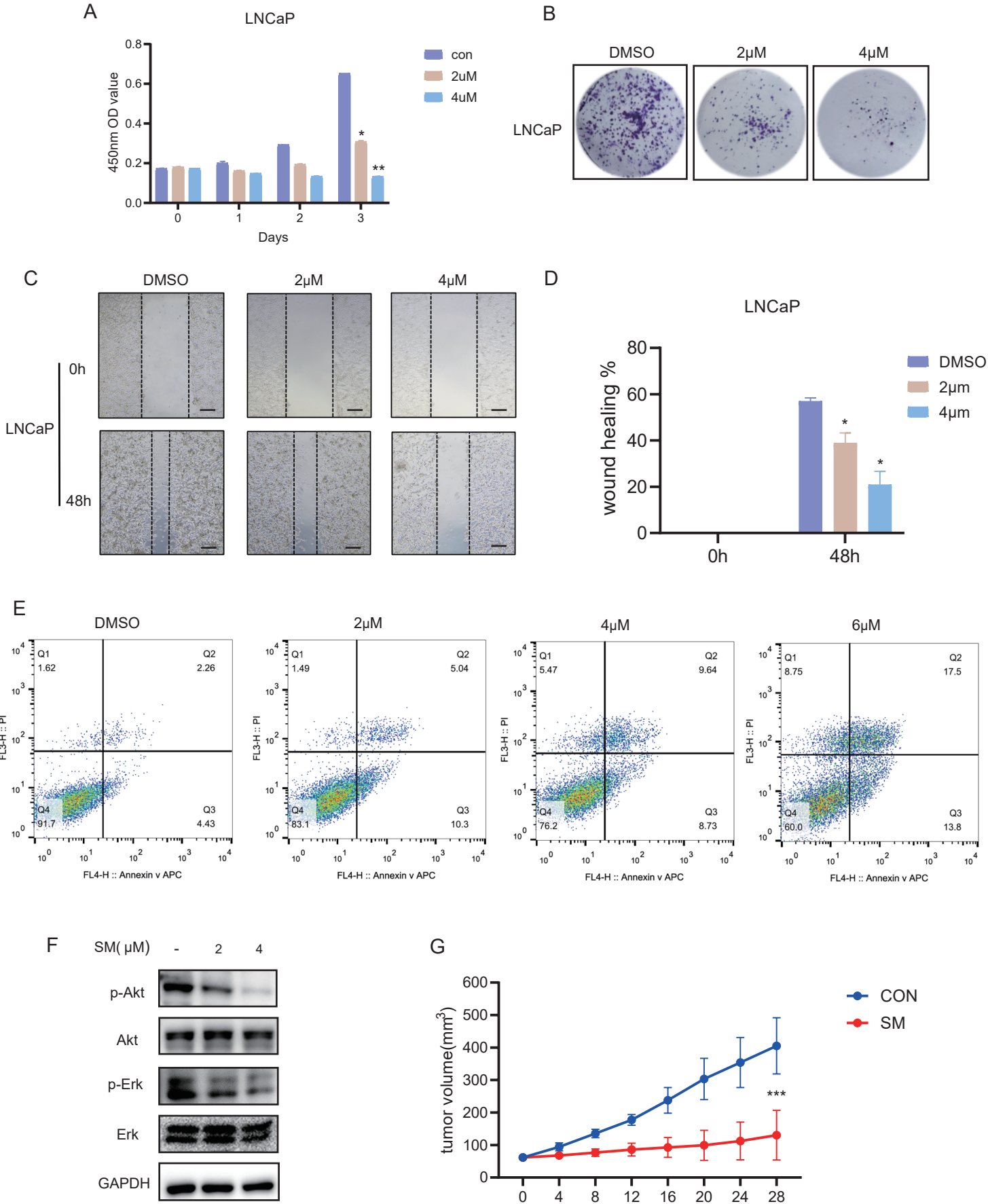

Supplement: Supplementary Materials — Figure S1: phenotype of AR-positive LNCaP cells induced by solamargine. [file 9055954.f1.pdf]
